# Commencement Exercises

**Published:** 1891-08

**Authors:** 


### COMMENCEMENTS.

### Louisville College of Dentistry.

The annual commencement exercises of the Louisville College of Dentistry, (Dental Department of the Central University of Kentucky) were held in connection with the exercises of the Medical Department, at Macauley's Theatre, Louisville, Ky., on Wednesday evening, June 17th, 1891.

A very interesting address was delivered by Prof. Francis Peabody on the history of the college and its progress, as well as of the profession generally.

The valedictory on behalf of the class was given by John T. Grant, of California.

The degree of D.D.S. was conferred on the following graduates by Rev. L. H. Blanton, D.D.S., Chancellor of the University:

| NAME. | NAME. |
|---|---|
| Otto B. Bachman. | William J. Botkin. |
| H. E. Cottingham. | George M. Dayton, M.D. |
| John C. Giltner. | Percy E. DeMille. |
| John T. Grant. | James P. Gray. |
| August W. Grubbel, Jr. | David S. Henry. |
| Elhanan M. Hight. | Abraham Howe, Jr. |
| James M. Johnston. | C. M. MacDonald. |
| Thaddeus J. J. Meder. | Benjamin T. Messick. |
| James Bashford Moore. | Charles Park Peters. |
| Chas. Walden Potter. | Wm. Sedwick Rogers. |
| Thomas B. Sanders. | Charles H. Sharp. |
| Chas. Filmore Ulmer. | Emmet W. Wagner. |
| Daniel W. Whipple. | John Haynes White. |

### University of California—College of Dentistry.

The ninth annual commencement exercises of the College of Dentistry of the University of California were held at Odd Fellow's Hall, San Francisco, California, Thursday November 13th, 1890, at eight o'clock P. M.

The number of matriculates was 63.

The degree of Doctor of Dental Surgery was conferred upon the following graduates by Martin Kellogg, A.M., President pro tem. of the University:

| NAME. | RESIDENCE. | NAME. | RESIDENCE. |
|---|---|---|---|
| Frederick H. Allbright | California. | Walter Romain Lovegrove | California. |
| Gotthard S. Backman | Sweden. | George Martin | California. |
| Frank Drake Burleson | California. | Clark Harrison Rawson | New York. |
| Paul Chas. Erhardt | California. | Richard McCargar | California. |
| William T. Heider | California. | John Matthew Redmond | California. |
| Charles Alexis Herrick | California. | David Warren Rulison | Nevada. |
| Saul Roberts Jacobs | California. | William Fuller Sharp | California. |
| Charles Ashby Litton | California. | Albert John Sylvester | California. |

### Boston Dental College.

The twenty-fourth annual commencement of this department was held in Berkeley Temple, June 17th, 1891, at 7:30 o'clock. An address was delivered by Rev. E. L. Rexford.

The number of matriculates for the year was 97.

The degree of D.D.S. was conferred on the following graduates by the President of the College I. J. Wetherbee, D.D.S.:

| NAME. | RESIDENCE. | NAME. | RESIDENCE. |
|---|---|---|---|
| Fred.. A. Barbour | New Brunswick. | Frederic S. Belyea | Massachusetts. |
| John B. Chandler | Massachusetts. | John G. Emerson | Massachusetts. |
| Edmund J. Ferry | Massachusetts. | Frank E. Follett | Maine. |
| Walter E. French | Massachusetts. | Jennie H. Gallap, | Rhode Island. |
| True A. Hoadley | Vermont. | Kristian N. E. Noeg | Norway. |
| Frank B. Johnston | Massachusetts. | Charles G. Lacaillard | Massachusetts. |
| Walter A. Lesure | Massachusetts. | Knut J. Luttropp | Massachusetts. |
| Frederic W. Lyons | Massachusetts. | Joseph T. M. Govern | Massachusetts. |
| William E. Nickerson | New York. | Ernest P. Peake | S. Africa. |
| Oliver Pease | Massachusetts. | Walter N. Pierce | New Hampshire. |
| Marc W. Pray | Massachusetts. | John S. Scott | P-Q. |
| Henry K. Shotswell | Massachusetts. | George A. Thatcher | Massachusetts. |
| Benjamin H. Thornton | Massachusetts. | Sherman E. Vinton | Rhode Island. |
| Asaph J. Walker | Maine. | Clarence H. Wall | Massachusetts. |
| Edwin D. Wood | New Brunswick. | Austin W. Woodman | New Hampshire. |
| Marion L. Woodward | France. | | |

### Harvard University—Dental Department.

The annual commencement of the Dental Department of the Harvard University was held in connection with the commencements of the other departments of that institution in Sanders Theatre on Wednesday, June 24th, 1891.

The degree of D.D.S. was conferred upon the following graduates by the President of the University Chas. W. Elliot, L.L.D.:

| NAME. | RESIDENCE. | NAME. | RESIDENCE. |
|---|---|---|---|
| Paul Boitel | Switzerland. | Clarence Moore Noble | Canada. |
| Georges Antoine Brouillet | France. | Hugh Owen | New Zealand. |
| Adin Albert Goldsmith (D.D.S. University of Pa) | New Hampshire. | Joseph Totten Paul | Boston. |
| Amos Irving Hadley | New Bedford. | George Barnum Perry | Illinois. |
| George Meads Holden | Lowell. | William Tuller Sharp, D.D.S., (University of California) | California. |
| Shimpei Nobustune Isawa | Japan. | Fred Homer Woodcock | Worcester. |
| George Martin (D.D.S. University of California) | California. | Alex. Humbolt Fisher | Boston. |

